# Correlation of Immunological and Histopathological Features with Gene Expression-Based Classifiers in Colon Cancer Patients

**DOI:** 10.3390/ijms232012707

**Published:** 2022-10-21

**Authors:** Simone van de Weerd, Marloes A. Smit, Jessica Roelands, Wilma E. Mesker, Davide Bedognetti, Peter J. K. Kuppen, Hein Putter, Rob A. E. M. Tollenaar, Jeanine M. L. Roodhart, Wouter Hendrickx, Jan Paul Medema, J. Han J. M. van Krieken

**Affiliations:** 1 Center for Experimental and Molecular Medicine, Cancer Center Amsterdam, Amsterdam UMC, University of Amsterdam, 1105 AZ Amsterdam, The Netherlands; 2 Department of Pathology, Radboud University Medical Centre, 6525 GA Nijmegen, The Netherlands; 3 Oncode Institute, Amsterdam UMC, University of Amsterdam, 3521 AL Amsterdam, The Netherlands; 4 Department of Surgery, Leiden University Medical Center, 2333 ZD Leiden, The Netherlands; 5 Translational Medicine Department, Research Branch, Sidra Medicine, Doha 26999, Qatar; 6 College of Health and Life Sciences, Hamad Bin Khalifa University, Qatar Foundation, Doha 34110, Qatar; 7 Department of Medical Statistics, Leiden University Medical Center, 2333 ZD Leiden, The Netherlands; 8 Department of Medical Oncology, University Medical Center Utrecht, Utrecht University, 3584 CX Utrecht, The Netherlands

**Keywords:** colon cancer, histopathology, consensus molecular subtypes, CRC intrinsic subtypes, tumor infiltrating lymphocytes, mucinous adenocarcinoma, tumor-stroma ratio, tumor budding

## Abstract

The purpose of this study was to evaluate the association between four distinct histopathological features: (1) tumor infiltrating lymphocytes, (2) mucinous differentiation, (3) tumor-stroma ratio, plus (4) tumor budding and two gene expression-based classifiers—(1) consensus molecular subtypes (CMS) plus (2) colorectal cancer intrinsic subtypes (CRIS). All four histopathological features were retrospectively scored on hematoxylin and eosin sections of the most invasive part of the primary tumor in 218 stage II and III colon cancer patients from two independent cohorts (AMC-AJCC-90 and AC-ICAM). RNA-based CMS and CRIS assignments were independently obtained for all patients. Contingency tables were constructed and a χ2 test was used to test for statistical significance. Odds ratios with 95% confidence intervals were calculated. The presence of tumor infiltrating lymphocytes and a mucinous phenotype (>50% mucinous surface area) were strongly correlated with CMS1 (*p* < 0.001 and *p* = 0.008) and CRIS-A (*p* = 0.006 and *p* < 0.001). The presence of mucus (≥ 10%) was associated with CMS3: mucus was present in 64.1% of all CMS3 tumors (*p* < 0.001). Although a clear association between tumor-stroma ratio and CMS4 was established in this study (*p* = 0.006), still 32 out of 61 (52.5%) CMS4 tumors were scored as stroma-low, indicating that CMS4 tumors cannot be identified solely based on stromal content. Higher budding counts were seen in CMS4 and CRIS-B tumors (*p* = 0.045 and *p* = 0.046). No other associations of the measured parameters were seen for any of the other CRIS subtypes. Our analysis revealed clear associations between histopathologic features and CMS or CRIS subtypes. However, identification of distinct molecular subtypes solely based on histopathology proved to be infeasible. Combining both molecular and morphologic features could potentially improve patient stratification.

## 1. Introduction

Colon cancer (CC) is a complex and heterogeneous disease with significant variation in therapy response and clinical outcome. The tumor-node-metastasis (TNM) classification, based on tumor extension and invasion, provides prognostic information and is currently used in clinical decision-making [1]. The backbone of treatment in patients with non-metastatic CC is surgical resection of the primary tumor. Adjuvant systemic chemotherapy is additionally applied in patients with high-risk stage II and stage III disease. Clinical outcome, however, still varies substantially between patients within the same TNM stage. Additional biomarkers and classification systems have been proposed to further stratify CC patients and improve prognostication beyond the TNM classification. These systems focus on different levels of tumor biology, including morphology of tumor cells and the tumor microenvironment, transcriptomic analysis, and microsatellite instability (MSI) status.

MSI tumors represent approximately 15% of all CC patients and are associated with favorable prognosis in early-stage disease, while worse prognosis is seen in metastatic CC as compared to microsatellite stable (MSS) CC [2,3,4]. MSI status is at present the only biomarker used in clinical practice with predictive value for adjuvant chemotherapy. Patients with MSI high-risk stage II CC should not be treated with adjuvant chemotherapy [5,6]. Two methods are commonly used in screening for MSI status: (1) polymerase chain reaction (PCR) testing of DNA mutation status for two to five microsatellite markers [7,8] and (2) immunohistochemical staining (IHC) for mismatch repair proteins (MLH1, MSH2, MSH6 and PMS2) [9]. Both methods are highly sensitive and specific, and the two tests show high concordance [10,11]. 

Stratification of CC patients by MSI status provides essential prognostic and predictive information but does not reflect the full complexity of the tumor and its interactions with the tumor microenvironment (TME). The TME plays an important role in cancer initiation and progression. Fibroblasts and macrophages are key players within the tumor stroma and secrete a variety of active factors such as cytokines, chemokines and growth factors that can regulate tumor occurrence and development and clinical outcome [12]. The composition of the TME varies substantially between CC patients. A high level of immune cell infiltration is present in a subset of tumors, while others demonstrate a high amount of stromal cells within their TME [13]. 

These differences in TME composition can in part be captured via assessment of routine hematoxylin and eosin (H&E)-stained tissue sections, which is relatively cheap and easy to implement in daily clinical practice. Two promising histopathological features are the presence and quantification of tumor infiltrating lymphocytes (TILs) and an estimation of the amount of stroma within the primary tumor. The importance of an intra-tumoral immune reaction as a prognostic marker is increasingly recognized, and a recent meta-analysis in CC patients established the improved overall survival for high levels of TILs as compared to low levels [14,15,16,17,18]. Tumors with a high amount of stroma (>50%) have an unfavorable prognosis compared to tumors with low stromal content (≤50%), and this observation has led to the development of the tumor-stroma ratio (TSR) as an indicator of clinical disease outcome [19,20,21]. 

Another morphologic characteristic of colon tumors is the absence or presence of extracellular mucus in the primary tumor. Mucinous adenocarcinomas, defined as tumors in which >50% of the lesion is composed of extracellular mucus, are considered a distinct histological subtype according to the World Health Organization [22]. Mucinous adenocarcinomas are associated with worse outcome compared to non-mucinous adenocarcinomas [23,24,25,26].

An important biomarker reflecting the invasive capacity of CC is the occurrence of tumor budding (TB). Tumor buds are defined as single cells or small clusters of up to four cells dissociated from the main tumor body [27]. Tumor buds are characterized by an epithelial-to-mesenchymal transition (EMT) phenotype and are considered to be an important step in cancer metastasis formation [28].

These distinct morphologic characteristics provide prognostic information and represent biological aspects of colon cancer (CC). In order to gain further insight into the exact underlying tumor biology, several research groups attempted to capture the entire tumor phenotype within gene expression profiles. These transcriptomic approaches aimed to gain further insight into the different aspects influencing tumor behavior. The CMS classifier, based on the entire tumor area (i.e., neoplastic cells as well as stromal cells), divides CC into four biologically distinct subtypes [29]. The clinical relevance of these biologic intrinsic processes implicated in each CMS was confirmed via CMS subtyping in a large heterogeneous patient cohort (*n* = 2129), which revealed significant differences in prognosis, with CMS4 as the poor-prognosis subtype [29].

The CRIS classification followed a different approach and focusses on the epithelial tumor compartment. Transcriptional profiles of patient-derived xenografts (PDXs) were used to define five cancer epithelium specific subtypes [30]. CRIS subtypes can be divided into two major subfamilies: CRIS-A/B and CRIS-C/D/E. CRIS-A and -B are both associated with mucinous and inflammatory traits, but CRIS-B as opposed to CRIS-A also displays marked traits of EMT. CRIS-C, -D and -E are characterized by chromosomal instability (CIN). Elevated EGFR signaling is seen in CRIS-C, while CRIS-D features high WNT activity. Lastly, CRIS-E features a Paneth cell-like phenotype. 

Certain prominent genotypic features of the molecular subtypes, such as an EMT phenotype in CMS4 tumors, are reminiscent of distinctive morphologic tumor traits, such as high stromal content. In this study, we assessed the association between four distinct histopathological features (TILs, mucinous differentiation, TSR and TB) and two gene expression-based classifiers (CMS and CRIS). Our aim was to evaluate whether these molecular and morphologic features, focusing on different levels of tumor biology, are interconnected or rather represent independent biomarkers.

## 2. Results

A total of 218 patients were retrospectively included in this study: 83 patients of the AMC-AJCC-90 cohort and 135 patients of the AC-ICAM cohort, of which 78 patients presented with stage II and 57 patients with stage III CC. Age, gender, and tumor sidedness did not differ between the cohorts (Table 1). The majority of tumors (86.7%) showed invasion through the muscularis propria (T3). 

CMS2 (33.9%) was the most prevalent subtype followed by CMS4 (28.0%). Within the CRIS classification, CRIS-A (27.5%) and CRIS-C (28.9%) were the most prevalent subtypes. No significant differences were seen in the distribution of CMS and CRIS subtypes between the cohorts (Table 1). 

Almost all CMS1 tumors were assigned to CRIS-A (46.5%) and CRIS-B (41.9%). CMS2 samples were partitioned into CRIS-C (60.8%), CRIS-D (17.6%), and CRIS-E (20.3%). CMS3 was predominantly assigned to CRIS-A (75.0%), and CMS4 was distributed across all five CRIS classes. This pattern of overlap is in line with previous results [30,31,32]. The distribution of the histopathological features within CMS and CRIS subtypes is shown in Table 2 and Table 3. 

A significant difference was seen in CMS distribution when stratifying for pathologic T-stage (*p* = 0.041). While the distribution of CMS1, -2, and -4 was similar, there was an enrichment for pT2 and pT4 tumors within CMS3, as compared to the other subtypes (Table S1). No significant differences were seen in the distribution of CRIS subtypes and histopathologic features per pathologic T-stage (Tables S2 and S3).

An overview of the calculated odds ratios between histopathological features and CMS subtypes and CRIS subtypes plus MSI status is depicted in Supplementary Tables S4–S6. 

### 2.1. Tumor Infiltrating Lymphocytes

**Figure 1:**
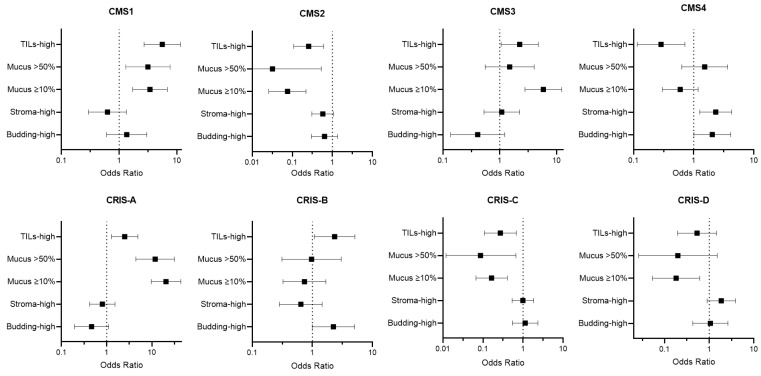

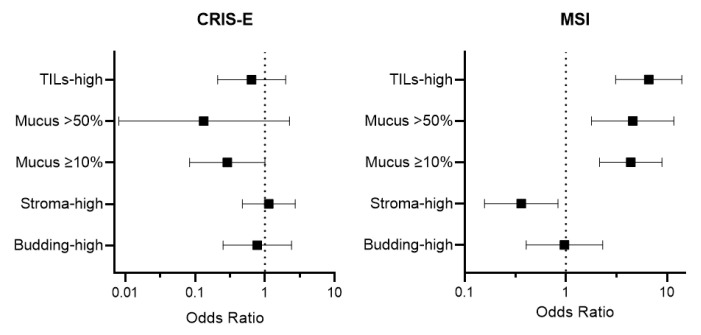
Odds ratios with 95% confidence interval calculated as the likelihood of classification into a certain molecular subtype (CMS, CRIS, or MSI) in the case of assignment into the highest histopathologic score category (i.e., TILs-high, mucus > 50%, mucus ≥ 10%, stroma-high and budding-high).

**Figure 2:**
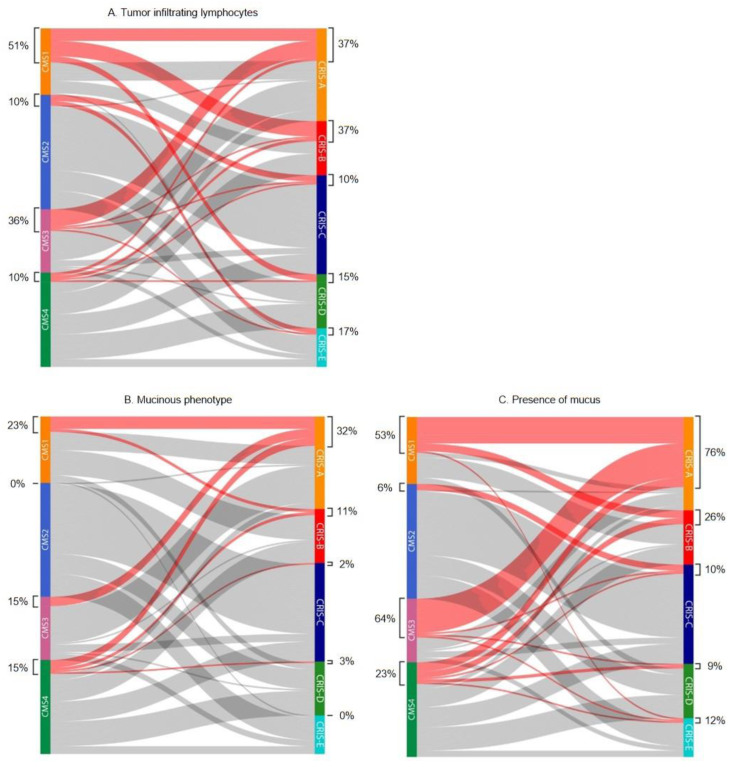

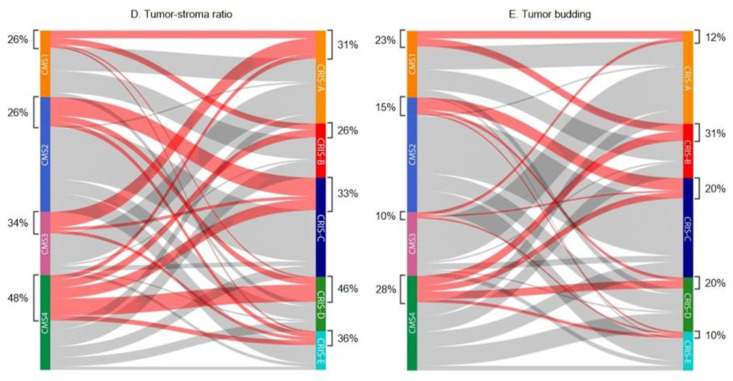
Sankey plots depicting the distribution of histopathological features within consensus molecular subtypes (CMS) and CRC intrinsic subtypes (CRIS). Percentages represent the number of tumors scored into a specific histopathologic category within CMS or CRIS subtypes. (**A**) Colored lines represent tumors with intermediate or high levels of tumor infiltrating lymphocytes. (**B**) Tumors with mucinous phenotype are highlighted. (**C**) Tumors containing any amount of mucus are depicted by colored lines. (**D**) Colored lines represent stroma-high tumors. (**E**) Budding-high tumors are highlighted.

TILs were scored as none/low in the majority of cases (77.1%). Intermediate and high levels of TILs were present in respectively 16.4% (*n* = 35) and 6.5% (*n* = 14). For odds ratio calculations, TILs were divided into two categories: none/low vs. intermediate/high. As expected, intermediate/high levels of TILs were significantly associated with CMS1 (OR 5.587 95% CI 2.704–11.543, *p* < 0.001) and to a lesser extent with CMS3 (OR 2.240 95% CI 1.056–4.751, *p* = 0.033) (Figure 1). Almost all CMS2 (65/72, 90.3%) and CMS4 (54/60, 90.0%) tumors contained a low number of TILs (Table 2 and Figure 2A).

Considering the CRIS classification, tumors with high levels of TILs were more likely to be assigned to CRIS-A and CRIS-B (OR 2.507 95% CI 1.280–4.908, *p* = 0.006 and OR 2.347 95% CI 1.079–5.010, *p* = 0.028) (Figure 1). The majority of tumors (105/120, 87.5%) assigned to the other subfamily (CRIS-C, -D, and -E) were scored as TILs-low (Table 3, Figure 2A).

### 2.2. Mucinous Differentiation

Mucinous adenocarcinoma was identified in 11.7% (*n* = 25) of all cases, in line with previously reported incidences [33,34]. Mucus (≥10%) was present in 30.8% (*n* = 66) of the tumors. 

Strikingly, no mucinous tumors were classified as CMS2, the most prevalent CMS subtype (Table 2, Figure 2B). Mucinous tumors were more often seen within CMS1 (OR 3.152 95% CI 1.302–7.628, *p* = 0.008) and CRIS-A (OR 11.796 95% CI 4.418–31.491, *p* < 0.001) (Figure 1), and 19 out of 25 (76.0%) mucinous tumors were classified as CRIS-A. 

When analyzing the amount of mucus in the categories present (≥10%) or absent (<10%), a strong association was seen with CMS3 (OR 5.836 2.779–12.256, *p* < 0.001) (Figure 1). Mucus was present in 64.1% (*n* = 25) of all CMS3 tumors, compared to 53.5% (*n* = 23) in CMS1, 5.6% in CMS2 (*n* = 4), and 23.3% (*n* = 14) in CMS4. Almost all mucus containing tumors classified as CMS3 were assigned to CRIS-A (Figure 2C). 

### 2.3. Tumor-Stroma Ratio

Stroma-high tumors were seen in 33.5% of all cases, which is in line with previous reported percentages [35,36]. A good inter-observer agreement was reached for scoring TSR in the AMC-AJCC-90 cohort between the two observers (SW and MS) with a Cohen’s kappa of 0.77. As expected, stroma-high tumors were significantly associated with CMS4 (OR 2.327 95% CI 1.263–4.289, *p* = 0.006) (Figure 1). Almost halve of the CMS4 tumors featured a high stromal content (Figure 2D). No associations were seen between TSR and CRIS subtypes, which is in line with our expectations, since the stromal compartment is not taken into account by the CRIS classifier. 

Stroma-high tumors were more frequently scored as budding-high as compared to stroma-low tumors (52.4% vs. 29.0%, OR 2.696 95% CI 1.356–5.362, *p* = 0.004). Comparing the histopathological features in any other combination did not reveal a significant association. 

### 2.4. Tumor Budding

Two categories were used for statistical analyses: TB-low (0–4 buds) and TB-high (≥5 buds). Tumors were categorized as TB-high in 19.3%. Higher percentages of TB-high tumors, ranging from 28% to 34%, have been reported in the literature [37,38]. These studies included patients with stages I–IV colorectal cancer. Higher tumor budding is known to be correlated with higher TNM stages and this could explain the lower percentage observed in our study [39]. TB-high tumors were more prevalent within CMS4 with an OR of 2.040 (95% CI 1.009–4.126, *p* = 0.045) (Figure 1). CMS3 and CRIS-A tumors showed a limited number of buds (Table 2 and Table 3, Figure 2E). A significant association was seen between TB-high tumors and assignment into CRIS-B (2.247 95% CI 0.998–5.059, *p* = 0.046). 

### 2.5. Combination of Histopathologic Features

An additional assessment was performed combining different histopathologic features to determine whether this would improve identification of CMS or CRIS subtypes. For this analysis, histopathologic features were selected that proved to be positively correlated with the same subtype: TILs and mucus for CMS1 and for CRIS-A, plus TSR and budding for CMS4.

First, tumors with both features present were analyzed compared to the rest. Because the histopathologic features showed limited overlap, a low number of tumors containing both features were selected. Combining high TILs and a mucinous phenotype resulted in just 6 cases. Twenty cases contained a high level of TILs and any amount of mucus. Both selections did not improve the identification of CMS1 or CRIS-A (Tables S7 and S8). Similarly, only 22 cases were scored as stroma-high and budding-high, which also did not improve the identification of CMS4 cases (Table S7).

As a next step, tumors that contained at least one of the positively correlated features were combined and compared to tumors without these features. This resulted in 68 (TILs-high or mucinous), 95 (TILs-high or mucus present) and 93 cases (stroma-high or budding high). Combining tumors with a high level of TILs or any amount of mucus, allowed for the identification of a higher number of CMS1 tumors. Separately, TILs and the presence of mucus correctly identified ~50% of CMS1 tumors, and this improved to 83.7% (36/43) when these features were combined (Table S7). However, this combination was clearly not specific for CMS1 and resulted in 59 (62.1%) false-positive cases. When combining TILs with a mucinous phenotype (>50% mucus), 69.8% (30/43) of CMS1 tumors were correctly identified (Table S7). However, this combination also led to a high number of false-positive cases (38/68).

The combination of stroma-high or budding high tumors, slightly improved the identification of CMS4 tumors, from 47.5% (stroma alone) to 59.0%. However, a high number of CMS1-3 tumors (*n* = 57) was incorrectly identified using this combination.

As mentioned before, the presence of mucus was strongly correlated with CRIS-A. Using this feature, 76.3% (45/59) of CRIS-A tumors were identified, and this could not be improved by using the combination of TILs or the presence of mucus (Table S8). 

### 2.6. Microsatellite Instability

MSI status was known for 194/218 (89.0%) tumors. In total, 44 out of 194 (22.7%) tumors were MSI. As expected, the majority of MSI tumors were classified as CMS1 (32/44, 72.7%). Within the CRIS classification, MSI tumors were divided into CRIS-A (23/44, 52.3%) and CRIS-B (16/44, 36.4%). 

We also analyzed the association between histopathological features and MSI status. We found a significant association between MSI status and three histopathological features: TILs, mucinous differentiation, and TSR. Tumors with a high number of TILs were more likely to be MSI (OR 6.571 95% CI 3.102–13.920, *p* < 0.001). More than halve of the MSI tumors (23/44, 52.3%) contained an intermediate or high number of TILs, as compared to 21/147 (14.3%) of MSS tumors (*p* < 0.001). Jenkins et al. assessed the presence of TILs in 1098 colorectal cancer patients and reported similar numbers for MSS and MSI tumors [40]. 

Mucinous adenocarcinomas were also associated with MSI status (OR 4.567 95% CI 1.789–11.655, *p* = 0.001). More than half (25/44, 56.8%) of the MSI tumors contained ≥10% of mucus as opposed to 23.1% (34/149) of MSS tumors, (*p* < 0.001). 

Stroma-high tumors were almost all classified as MSS (57/65, 87.7%). Only 8/44 (18.2%) MSI tumors were scored as stroma-high. This is significantly lower than the 57/150 (38.0%) of MSS tumors (*p* = 0.014). No associations were seen between tumor budding and MSI status.

## 3. Discussion

Several histopathological features, such as tumor infiltrating lymphocytes (TILs), mucinous differentiation, the tumor-stroma ratio (TSR), and tumor budding (TB), are under development or currently used in clinical practice to improve patient stratification. These distinct morphologic characteristics provide prognostic information and are able to capture some biological aspects of colon cancer (CC). To gain further insight into the exact underlying tumor biology, focus has extended to CC stratification based on the tumor transcriptome, which led to the development of the CMS and CRIS subtypes. 

In this study, we assessed the association between four distinct histopathological features and two gene expression-based classifiers in 218 stage II and III colon cancer patients across two independent cohorts. In addition, we evaluated the adequacy of these features to identify CMS and CRIS subtypes. Our analyses revealed multiple significant associations between histopathological features and molecular subtypes. These associations were, however, not strong enough to adequately identify CMS or CRIS subtypes with histopathology alone. 

The effect of the immune infiltrate on tumor behavior and progression depends on a variety of factors, including the type of tumor and the specific cellular composition of the infiltrate and its intratumoral localization [41]. In this study, we quantified the level of intraepithelial TILs and compared this between different molecular subtypes. The presence and quantity of TILs was positively correlated with CMS1, in line with previous observations [29,42,43,44,45]. CMS1 is known to be enriched for microsatellite instability (MSI), which we could confirm in this study with 32 out of 40 (80.0%) CMS1 tumors being classified as MSI. Deficient mismatch repair status causes high levels of mutations and subsequent neo-antigen formation, which induces an immune response [29,46,47]. These observations could explain this particular genotype-phenotype correlation. In addition, the number of TILs has previously been shown to have value in predicting MSI status [40,48,49,50,51]. 

In contrast to our observations, previous studies identified two CMS subtypes with high expression of immune signatures: CMS1, as one would expect, and CMS4 [43,44]. Both studies used bulk RNA expression data to evaluate the repertoire of tumor infiltrating immune cells of each CMS. In contrast to our study, this method does not consider the intratumoral localization of the different immune cells as well as intra-epithelial as stromal immune cells were included. Next to this, both studies described the immune landscape of CMS4 as immune-inflamed and pro-tumoral, with high expression of immunosuppressive cells, such as M2 macrophages and regulatory T-cells. This specific immune landscape hampers activation of an adaptive immune response and might cause exclusion of TILs. These observations could well explain the low number of TILs within CMS4 tumors in our study. 

The near absence of TILs within CMS2 tumors as shown in our study is in line with current data. CMS2 tumors show low expression of genes implicated in T-cell chemotaxis and activation and poor expression of PD-1 and PD-L1 [29,43,44]. Accordingly, the CMS2 subtype is often referred to as an ‘immune desert’. Since CMS2 tumors account for approximately 37% of colon tumors, it is highly relevant to increase our understanding of this immune avoidance, especially in the context of the development of immunotherapies. 

Mucinous adenocarcinomas, defined as tumors in which >50% of the lesion is composed of extracellular mucus, constitute a distinct histologic subtype and are associated with *BRAF* mutations, MSI status, and the CpG island methylator phenotype (CIMP) [52]. These findings imply that mucinous adenocarcinomas are likely associated with CMS1. In agreement with this hypothesis, we identified a higher prevalence of mucinous tumors within CMS1: 23.3% (10 out of 43), as opposed to 0.0%, 15.4%, and 15.0% within CMS2, -3, and -4, respectively. Notably, no mucinous adenocarcinomas were classified as CMS2, which is the most prevalent CMS subtype. The substantially lower rate of mucinous tumors within CMS2 was confirmed in a retrospective review of pathology reports in 608 colorectal cancer patients [24] and in a recent study using a deep learning algorithm on digital slides to assess the extracellular mucin-to-tumor area [25]. Taken together, these results indicate a distinct biological background for mucinous adenocarcinomas compared to the classical CMS2 subtype.

Next to the 50% cut-off, we additionally categorized the amount of mucus as absent (<10%) or present (≥10%) in our analyses. Interestingly, the presence of mucus was strongly associated with CMS3. Although both CMS3 and mucinous tumors are known to be associated with *KRAS* mutations, the exact underlying mechanism for this association remains unclear [52]. Elucidating this mechanism might be of clinical importance, since it was recently reported that a mucinous phenotype significantly impairs 5-year overall survival within CMS3 tumors [25]. 

In line with our hypothesis and previous observations, both stroma-high and budding-high tumors were significantly associated with CMS4 [37,45,53]. Although a clear association between TSR and CMS4 was established, still 32 out of 61 (52.5%) CMS4 tumors were scored as stroma-low. Sandberg et al. reported similar numbers with 10 out of 18 (55.6%) CMS4 tumors scored as stroma-low [54]. Importantly, these results clearly indicate that CMS4, the poor-prognosis subtype, cannot be identified solely based on stromal content. 

Not much is known about the histopathologic characteristics of the CRIS subtypes. Isella et al. reported a high prevalence of mucinous tumors within CRIS-A and inflammatory traits, as defined via gene expression, within the CRIS-A and -B subfamily [30]. These observations were confirmed in this study, with a striking association between CRIS-A and a mucinous phenotype. No associations were seen between TSR plus TB and CRIS subtypes, except for a higher budding count in CRIS-B (*p* = 0.046).

Our thorough assessment of four distinct histopathologic features revealed clear associations between tumor morphology and CMS or CRIS subtypes. However, identification of these distinct molecular subtypes solely based on histopathology proved to be infeasible. The most potent marker was the quantification of TILs. Within the TILs-high category, 11 out of 14 tumors were classified as CMS1, resulting in a positive predictive value of 78.6%. However, 32 out of 43 CMS1 tumors would be misclassified using this criterion. In addition, CMS4 tumors are clearly associated with high stromal content. However, 44 out of 73 (60%) stroma-high tumors are classified as CMS1–3. Similar numbers are seen with the presence of mucus, which is strongly associated with CMS3. If mucus is present in a tumor, 62% are classified as CMS1, -2, or -4. 

Combining both molecular and morphologic features could potentially improve patient stratification. For example, response to immunotherapy within CMS1 tumors might be different for tumors with a high number of TILs as compared to tumors with a low number of TILs. It would be of interest to evaluate the clinical relevance of combining CMS or CRIS subtypes with histopathologic markers. 

An important strength of this study is the consistent scoring method of four different histopathological features in two independent cohorts for which the gold standard RNA-based CMS and CRIS assignments were available. Our analyses are however potentially influenced by tumor heterogeneity, since the tumor area used for histopathologic assessment differs from the region sampled for RNA extraction and subsequent molecular classification. Furthermore, the use of more advanced scoring methods like immunohistochemistry and digital pathology might have extended our findings, but we were particularly interested in the value of routine histopathology. 

As is well known, colon cancer is a complex and heterogeneous disease driven by both genetic alterations and the host response. Tumor behavior is highly influenced by the microenvironment, which is composed of varying cell types with immune cells and stromal cells as key players. Each feature described in this study focusses on a different level of tumor biology. The number of TILs and amount of stroma mainly relates to the TME, while tumor budding and the CRIS classification focus on the epithelial tumor compartment. Mucinous differentiation and the CMS classification are based on the entire tumor compartment. 

Some characteristics are shared between the molecular subtypes and histopathologic features. Our thorough assessment of four different histopathological features indeed allowed us to confirm several previously reported associations and identify new genotype-phenotype correlations. Identification of distinct molecular subtypes solely based on histopathology proved to be infeasible. Larger studies are needed to evaluate whether combining both molecular and morphologic features could improve the identification of CC patients that would benefit from systemic treatment, such as chemotherapy or immunotherapy. 

## 4. Materials and Methods

### 4.1. Patient Cohorts

Two independent patient cohorts were used in this study. The AMC-AJCC-90 cohort consists of stage II colon cancer patients that underwent intentionally curative surgery between 1997 and 2006 (GSE33113) [55]. The second cohort (AC-ICAM) contains stage II and III colon cancer patients who underwent surgery at Leiden University Medical Centre, the Netherlands, between 2001 and 2015 [56] and were transcriptomically profiled in Sidra Medicine, Qatar. No rectal cancers were included. Patients with available formalin-fixed paraffin-embedded (FFPE) H&E sections and gene expression data were included in this study.

### 4.2. CMS and CRIS Classification

CMS classification was determined on microarray (AMC-AJCC-90 cohort) or mRNAseq gene expression data (AC-ICAM cohort) using the random forest CMS classifier [29]. Nearest CMS labels were used. Patients from both cohorts were classified into CRIS subtypes using the ‘CRIS classifier’ package provided by Isella et al. [30]. 

### 4.3. MSI Status

MSI status was previously determined in both cohorts using two different methods. The AMC-AJCC-90 samples were analyzed using the MSI Analysis System, version 1.2 (Promega, Madison, WI, USA), according to the manufacturer’s instructions. Samples were considered MSI when more than one out of five markers were instable. In the AC-ICAM cohort, MSI status was determined on whole exome sequencing (WES) data using MANTIS (v.1.0.4), a tool for rapid detection of microsatellite instability [57]. Briefly, MANTIS calculates and compares the instability scores of microsatellite regions between tumors and a matched reference genome. Samples are classified as MSI-H (MANTIS score >0.4) or MSS (MANTIS score ≤0.4). 

### 4.4. Ethical Considerations

All research activities were performed with coded-anonymous tissue samples and data. All data and patient material were handled in accordance with the 1964 Helsinki Declaration and its later amendments and the Code of conduct. Informed consent was obtained from all patients. The use of patient data and tissue were approved by the ethics/institutional review board at the respective institutions. 

### 4.5. Histopathological Features

All histopathological features were scored on FFPE H&E sections from the most invasive tumor part of the primary tumor. The observers were blinded for clinicopathological data and the CMS and CRIS classification. The TSR was previously scored according to recommendations in the AC-ICAM cohort [20,58]. Within the AJCC-AMC90 cohort, two observers (MS and SW) scored the TSR and a third observer (HK) was consulted in case of disagreement. All other histopathologic biomarkers were scored by one observer (HK). 

#### 4.5.1. Tumor Infiltrating Lymphocytes

**Figure 3:**
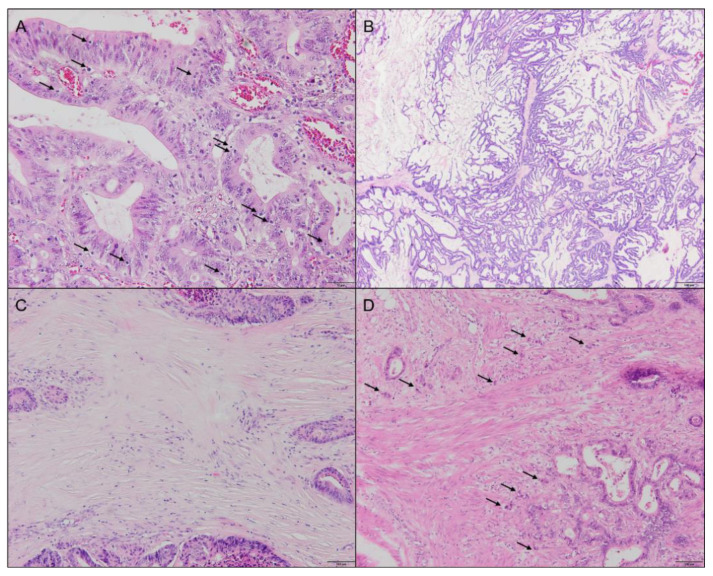
Examples of hematoxylin and eosin-stained sections representative for different histopathologic categories. (**A**) Tumor with high amount of tumor infiltrating lymphocytes, indicated by arrows, 20× objective. (**B**) Mucinous tumor, 2× objective. (**C**) Stroma-high tumor, 10× objective. (**D**) Tumor scored as budding-high, arrows indicate tumor buds, 10× objective.

Tumor infiltrating lymphocytes (TILs) were scored as an average count of lymphocytes present in the tumor epithelium (not the stromal compartment) within the borders of the invasive tumor at 200× magnification. TILs were scored in three categories vis eye-balling: none/low, intermediate, and high. Figure 3A shows an example of a TILs-high tumor. 

#### 4.5.2. Mucinous Differentiation

The presence of extracellular mucus was scored in percentages with 10% increments for the whole tumor area. For the analysis, tumors were categorized as mucus absent (<10%) versus present (≥10%) and mucinous (>50%) versus non-mucinous (≤50%) carcinoma. An example of a mucinous tumor is depicted in Figure 3B. 

#### 4.5.3. Tumor-Stroma Ratio

The tumor-stroma ratio was scored as previously described [20]. In short, areas containing the highest amount of stroma were selected. Next, the percentage of stroma was scored in 10% increments in one vision site, in which tumor cells were present at all borders using a 10× objective. Stroma-high was defined as >50% of stromal area and stroma-low as ≤50% stromal area. Figure 3C represents an example of a stroma-high tumor. 

#### 4.5.4. Tumor Budding

Tumor buds were defined as single cells or small clusters of up to four cells dissociated from the main tumor body. TB was scored at the invasive front within a single vision field using a 20× objective, according to the consensus recommendations [27]. Due to the small number of patients in each TB group, we combined the intermediate and high groups into one category for statistical analysis. An example of a TB-high tumor is shown in Figure 3D. 

### 4.6. Statistical Analysis

Continuous data were compared using an independent *t*-test and are shown as mean and standard deviation. Categorical patient and tumor characteristics were compared using a χ^2^ test. Contingency tables were constructed to analyze the association between histopathologic markers and CMS or CRIS subtypes. χ^2^ test was used to test for statistical significance. Fisher’s exact test was used in case one or more cells had a value of zero. Odds ratios (OR) with 95% confidence intervals were calculated. Haldane correction was used to avoid errors in OR calculations when one or more cells in a data table had a value of zero. This correction adds 0.5 to all cells. Cohen’s kappa coefficient was used to determine the interobserver agreement. *p*-values are two-tailed and results <0.05 are considered significant. Statistical analyses were conducted in IBM SPSS software version 28 (SPSS, Inc. an IBM Company Chicago, IL, USA).

## Figures and Tables

**Table 1:** Baseline characteristics of the included cohorts. Age is depicted as mean with standard deviation, other characteristics as absolute number and percentage. *p*-values are derived from comparison between the AMC-AJCC-90 and AC-ICAM cohort.

		Combined	AMC-AJCC-90	AC-ICAM	
		(*n* = 218)	(*n* = 83)	(*n* = 135)	*p*-Value
**Age**					
	Mean (SD)	69.2 (11.8)	69.5 (13.1)	69.1 (10.9)	0.818
**Gender**					
	Male	112 (51.4)	38 (45.8)	74 (54.8)	0.195
	Female	106 (48.6)	45 (54.2)	61 (45.2)	
**Localization**					
	Right	113 (51.8)	44 (53.0)	69 (51.1)	0.785
	Left	105 (48.2)	39 (47.0)	66 (48.9)	
**pT stage**					
	2	7 (3.2)	0 (0)	7 (5.2)	0.103
	3	189 (86.7)	75 (90.4)	114 (84.4)	
	4	22 (10.1)	8 (9.6)	14 (10.4)	
**CMS**					
	1	43 (19.7)	20 (24.1)	23 (17.0)	0.310
	2	74 (33.9)	31 (37.3)	43 (31.9)	
	3	40 (18.3)	12 (14.5)	28 (20.7)	
	4	61 (28.0)	20 (24.1)	41 (30.4)	
**CRIS**					
	A	60 (27.5)	26 (31.3)	34 (25.2)	0.754
	B	35 (16.1)	12 (14.5)	23 (17.0)	
	C	63 (28.9)	21 (25.3)	42 (31.1)	
	D	35 (16.1)	15 (18.1)	20 (14.8)	
	E	25 (11.5)	9 (10.8)	16 (11.9)	

**Table 2:** Distribution of histopathologic features within the consensus molecular subtypes, shown as number and percentage. TILs = tumor infiltrating lymphocytes, TSR = tumor-stroma ratio. *p*-values are derived from an overall comparison between subtypes.

		CMS1	CMS2	CMS3	CMS4	*p*-Value
**TILs**						
	None/low	21 (48.8)	65 (90.3)	25 (64.1)	54 (90.0)	<0.001
	Intermediate	11 (25.6)	7 (9.7)	12 (30.8)	5 (8.3)	
	High	11 (25.6)	0 (0)	2 (5.1)	1 (1.7)	
**Mucus**						
	≤50%	33 (76.7)	72 (100)	33 (84.6)	51 (85.0)	0.001
	>50%	10 (23.3)	0 (0)	6 (15.4)	9 (15.0)	
**Mucus**						
	<10%	20 (46.5)	68 (94.4)	14 (35.9)	46 (76.7)	<0.001
	≥10%	23 (53.5)	4 (5.6)	25 (64.1)	14 (23.3)	
**TSR**						
	Stroma-low	32 (74.4)	55 (74.3)	26 (65.0)	32 (52.5)	0.034
	Stroma-high	11 (25.6)	19 (25.7)	14 (35.0)	29 (47.5)	
**Tumor Budding**						
	Low (<5)	33 (76.7)	63 (85.1)	36 (90.0)	44 (72.1)	0.271
	Intermediate (5–9)	7 (16.3)	7 (9.5)	2 (5.0)	13 (21.3)	
	High (≥10)	3 (7.0)	4 (5.4)	2 (5.0)	4 (6.6)	

**Table 3:** Distribution of histopathologic features within the CRC intrinsic subtypes, shown as number and percentage. TILs = tumor infiltrating lymphocytes, TSR = tumor stroma ratio. *p*-values are derived from an overall comparison between subtypes.

		CRIS-A	CRIS-B	CRIS-C	CRIS-D	CRIS-E	*p*-Value
**TILs**							
	None/low	38 (64.4)	22 (62.9)	56 (90.3)	29 (85.3)	20 (83.3)	0.001
	Intermediate	17 (28.8)	7 (20.0)	6 (9.7)	2 (5.9)	3 (12.5)	
	High	4 (6.8)	6 (17.1)	0 (0)	3 (8.8)	1 (4.2)	
**Mucus**							
	≤50%	40 (67.8)	31 (88.6)	61 (98.4)	33 (97.1)	24 (100)	<0.001
	>50%	19 (32.2)	4 (11.4)	1 (1.6)	1 (2.9)	0 (0)	
**Mucus**							
	<10%	14 (23.7)	26 (74.3)	56 (90.3)	31 (91.2)	21 (87.5)	<0.001
	≥10%	45 (76.3)	9 (25.7)	6 (9.7)	3 (8.8)	3 (12.5)	
**TSR**							
	Stroma-low	42 (70.0)	26 (74.3)	42 (66.7)	19 (54.3)	16 (64.0)	0.448
	Stroma-high	18 (30.0)	9 (25.7)	21 (33.3)	16 (45.7)	9 (36.0)	
**Tumor Budding**							
	Low (<5)	53 (88.3)	24 (68.6)	50 (79.4)	28 (80.0)	21 (84.0)	0.429
	Intermediate (5–9)	4 (6.7)	9 (25.7)	9 (14.3)	5 (14.3)	2 (8.0)	
	High (≥10)	3 (5.0)	2 (5.7)	4 (6.3)	2 (5.7)	2 (8.0)	

## Data Availability

The datasets used and/or analyzed during the current study are available from the corresponding author on reasonable request.

## Supplementary Materials

The following supporting information can be downloaded at: https://www.mdpi.com/article/10.3390/ijms232012707/s1. Table S1: Distribution of CMS subtypes per pathologic T-stage. Table S2: Distribution of CRIS subtypes per pathologic T-stage. Table S3: Correlation between histopathologic features and pathologic T-stage. Table S4: Odds ratios between histopathological features and CMS subtypes. Table S5: Odds ratios between histopathological features and CRIS subtypes. Table S6: Odds ratios between histopathological features and MSI status. Table S7: Correlation between different combinations of histopathologic features and CMS subtypes. Table S8: Correlation between different combinations of histopathologic features and CRIS subtypes.

## References

[1] Amin M.B., Greene F.L., Edge S.B., Compton C.C., Gershenwald J.E., Brookland R.K., Meyer L., Gress D.M., Byrd D.R., Winchester D.P. (2017). The Eighth Edition AJCC Cancer Staging Manual: Continuing to build a bridge from a population-based to a more “personalized” approach to cancer staging. CA Cancer J. Clin..

[2] Boland C.R., Goel A. (2010). Microsatellite instability in colorectal cancer. Gastroenterology.

[3] Sinicrope F.A., Foster N.R., Thibodeau S.N., Marsoni S., Monges G., Labianca R., Kim G.P., Yothers G., Allegra C., Moore M.J. (2011). DNA mismatch repair status and colon cancer recurrence and survival in clinical trials of 5-fluorouracil-based adjuvant therapy. J. Natl. Cancer Inst..

[4] Sargent D.J., Marsoni S., Monges G., Thibodeau S.N., Labianca R., Hamilton S.R., French A.J., Kabat B., Foster N.R., Torri V. (2010). Defective mismatch repair as a predictive marker for lack of efficacy of fluorouracil-based adjuvant therapy in colon cancer. J. Clin. Oncol. Off. J. Am. Soc. Clin. Oncol..

[5] Aasebø K., Dragomir A., Sundström M., Mezheyeuski A., Edqvist P.H., Eide G.E., Ponten F., Pfeiffer P., Glimelius B., Sorbye H. (2019). Consequences of a high incidence of microsatellite instability and BRAF-mutated tumors: A population-based cohort of metastatic colorectal cancer patients. Cancer Med..

[6] Kim C.G., Ahn J.B., Jung M., Beom S.H., Kim C., Kim J.H., Heo S.J., Park H.S., Kim J.H., Kim N.K. (2016). Effects of microsatellite instability on recurrence patterns and outcomes in colorectal cancers. Br. J. Cancer.

[7] Venderbosch S., Nagtegaal I.D., Maughan T.S., Smith C.G., Cheadle J.P., Fisher D., Kaplan R., Quirke P., Seymour M.T., Richman S.D. (2014). Mismatch repair status and BRAF mutation status in metastatic colorectal cancer patients: A pooled analysis of the CAIRO, CAIRO2, COIN, and FOCUS studies. Clin. Cancer Res. Off. J. Am. Assoc. Cancer Res..

[8] Umar A., Boland C.R., Terdiman J.P., Syngal S., de la Chapelle A., Rüschoff J., Fishel R., Lindor N.M., Burgart L.J., Hamelin R. (2004). Revised Bethesda Guidelines for hereditary nonpolyposis colorectal cancer (Lynch syndrome) and microsatellite instability. J. Natl. Cancer Inst..

[9] Suraweera N., Duval A., Reperant M., Vaury C., Furlan D., Leroy K., Seruca R., Iacopetta B., Hamelin R. (2002). Evaluation of tumor microsatellite instability using five quasimonomorphic mononucleotide repeats and pentaplex PCR. Gastroenterology.

[10] Lindor N.M., Burgart L.J., Leontovich O., Goldberg R.M., Cunningham J.M., Sargent D.J., Walsh-Vockley C., Petersen G.M., Walsh M.D., Leggett B.A. (2002). Immunohistochemistry versus microsatellite instability testing in phenotyping colorectal tumors. J. Clin. Oncol. Off. J. Am. Soc. Clin. Oncol..

[11] Zhang X., Li J. (2013). Era of universal testing of microsatellite instability in colorectal cancer. World J. Gastrointest. Oncol..

[12] Hissong E., Crowe E.P., Yantiss R.K., Chen Y.T. (2018). Assessing colorectal cancer mismatch repair status in the modern era: A survey of current practices and re-evaluation of the role of microsatellite instability testing. Mod. Pathol..

[13] Asif P.J., Longobardi C., Hahne M., Medema J.P. (2021). The Role of Cancer-Associated Fibroblasts in Cancer Invasion and Metastasis. Cancers.

[14] Quante M., Varga J., Wang T.C., Greten F.R. (2013). The gastrointestinal tumor microenvironment. Gastroenterology.

[15] Marliot F., Pagès F., Galon J. (2020). Usefulness and robustness of Immunoscore for personalized management of cancer patients. Oncoimmunology.

[16] Idos G.E., Kwok J., Bonthala N., Kysh L., Gruber S.B., Qu C. (2020). The Prognostic Implications of Tumor Infiltrating Lymphocytes in Colorectal Cancer: A Systematic Review and Meta-Analysis. Sci. Rep..

[17] Pagès F., Mlecnik B., Marliot F., Bindea G., Ou F.S., Bifulco C., Lugli A., Zlobec I., Rau T.T., Berger M.D. (2018). International validation of the consensus Immunoscore for the classification of colon cancer: A prognostic and accuracy study. Lancet.

[18] Galon J., Mlecnik B., Bindea G., Angell H.K., Berger A., Lagorce C., Lugli A., Zlobec I., Hartmann A., Bifulco C. (2014). Towards the introduction of the ‘Immunoscore’ in the classification of malignant tumours. J. Pathol..

[19] Pagès F., Kirilovsky A., Mlecnik B., Asslaber M., Tosolini M., Bindea G., Lagorce C., Wind P., Marliot F., Bruneval P. (2009). In situ cytotoxic and memory T cells predict outcome in patients with early-stage colorectal cancer. J. Clin. Oncol. Off. J. Am. Soc. Clin. Oncol..

[20] van Pelt G.W., Sandberg T.P., Morreau H., Gelderblom H., van Krieken J., Tollenaar R., Mesker W.E. (2018). The tumour-stroma ratio in colon cancer: The biological role and its prognostic impact. Histopathology.

[21] van Pelt G.W., Kjaer-Frifeldt S., van Krieken J., Al Dieri R., Morreau H., Tollenaar R., Sorensen F.B., Mesker W.E. (2018). Scoring the tumor-stroma ratio in colon cancer: Procedure and recommendations. Virchows Arch..

[22] Huijbers A., Tollenaar R.A., v Pelt G.W., Zeestraten E.C., Dutton S., McConkey C.C., Domingo E., Smit V.T., Midgley R., Warren B.F. (2013). The proportion of tumor-stroma as a strong prognosticator for stage II and III colon cancer patients: Validation in the VICTOR trial. Ann. Oncol. Off. J. Eur. Soc. Med. Oncol..

[23] Nagtegaal I.D., Odze R.D., Klimstra D., Paradis V., Rugge M., Schirmacher P., Washington K.M., Carneiro F., Cree I.A. (2020). The 2019 WHO classification of tumours of the digestive system. Histopathology.

[24] Verhulst J., Ferdinande L., Demetter P., Ceelen W. (2012). Mucinous subtype as prognostic factor in colorectal cancer: A systematic review and meta-analysis. J. Clin. Pathol..

[25] Khan M., Loree J.M., Advani S.M., Ning J., Li W., Pereira A.A.L., Lam M., Raghav K., Morris V.K., Broaddus R. (2018). Prognostic Implications of Mucinous Differentiation in Metastatic Colorectal Carcinoma Can Be Explained by Distinct Molecular and Clinicopathologic Characteristics. Clin. Color. Cancer.

[26] Nguyen H.G., Lundström O., Blank A., Dawson H., Lugli A., Anisimova M., Zlobec I. (2021). Image-based assessment of extracellular mucin-to-tumor area predicts consensus molecular subtypes (CMS) in colorectal cancer. Mod. Pathol..

[27] Wang L., Hirano Y., Heng G., Ishii T., Kondo H., Hara K., Obara N., Asari M., Kato T., Yamaguchi S. (2020). Mucinous Adenocarcinoma as a High-risk Factor in Stage II Colorectal Cancer: A Propensity Score-matched Study from Japan. Anticancer. Res..

[28] Lugli A., Kirsch R., Ajioka Y., Bosman F., Cathomas G., Dawson H., El Zimaity H., Flejou J.F., Hansen T.P., Hartmann A. (2017). Recommendations for reporting tumor budding in colorectal cancer based on the International Tumor Budding Consensus Conference (ITBCC) 2016. Mod. Pathol..

[29] De Smedt L., Palmans S., Andel D., Govaere O., Boeckx B., Smeets D., Galle E., Wouters J., Barras D., Suffiotti M. (2017). Expression profiling of budding cells in colorectal cancer reveals an EMT-like phenotype and molecular subtype switching. Br. J. Cancer.

[30] Guinney J., Dienstmann R., Wang X., de Reynies A., Schlicker A., Soneson C., Marisa L., Roepman P., Nyamundanda G., Angelino P. (2015). The consensus molecular subtypes of colorectal cancer. Nat. Med..

[31] Isella C., Brundu F., Bellomo S.E., Galimi F., Zanella E., Porporato R., Petti C., Fiori A., Orzan F., Senetta R. (2017). Selective analysis of cancer-cell intrinsic transcriptional traits defines novel clinically relevant subtypes of colorectal cancer. Nat. Commun..

[32] Allen W.L., Dunne P.D., McDade S., Scanlon E., Loughrey M., Coleman H., McCann C., McLaughlin K., Nemeth Z., Syed N. (2018). Transcriptional subtyping and CD8 immunohistochemistry identifies poor prognosis stage II/III colorectal cancer patients who benefit from adjuvant chemotherapy. JCO Precis. Oncol..

[33] Alderdice M., Richman S.D., Gollins S., Stewart J.P., Hurt C., Adams R., McCorry A.M., Roddy A.C., Vimalachandran D., Isella C. (2018). Prospective patient stratification into robust cancer-cell intrinsic subtypes from colorectal cancer biopsies. J. Pathol..

[34] Leopoldo S., Lorena B., Cinzia A., Gabriella D.C., Angela Luciana B., Renato C., Antonio M., Carlo S., Cristina P., Stefano C. (2008). Two subtypes of mucinous adenocarcinoma of the colorectum: Clinicopathological and genetic features. Ann. Surg. Oncol..

[35] Glasgow S.C., Yu J., Carvalho L.P., Shannon W.D., Fleshman J.W., McLeod H.L. (2005). Unfavourable expression of pharmacologic markers in mucinous colorectal cancer. Br. J. Cancer.

[36] Mesker W.E., Liefers G.J., Junggeburt J.M., van Pelt G.W., Alberici P., Kuppen P.J., Miranda N.F., van Leeuwen K.A., Morreau H., Szuhai K. (2009). Presence of a high amount of stroma and downregulation of SMAD4 predict for worse survival for stage I-II colon cancer patients. Anal. Cell. Pathol..

[37] Mesker W.E., Junggeburt J.M., Szuhai K., de Heer P., Morreau H., Tanke H.J., Tollenaar R.A. (2007). The carcinoma-stromal ratio of colon carcinoma is an independent factor for survival compared to lymph node status and tumor stage. Anal. Cell. Pathol..

[38] Trinh A., Ladrach C., Dawson H.E., Ten Hoorn S., Kuppen P.J.K., Reimers M.S., Koopman M., Punt C.J.A., Lugli A., Vermeulen L. (2018). Tumour budding is associated with the mesenchymal colon cancer subtype and RAS/RAF mutations: A study of 1320 colorectal cancers with Consensus Molecular Subgroup (CMS) data. Br. J. Cancer.

[39] Lang-Schwarz C., Melcher B., Haumaier F., Lang-Schwarz K., Rupprecht T., Vieth M., Sterlacci W. (2018). Budding and tumor-infiltrating lymphocytes—Combination of both parameters predicts survival in colorectal cancer and leads to new prognostic subgroups. Hum Pathol..

[40] Zlobec I., Dawson H.E., Blank A., Bokhorst J.M., Berger M.D., Nagtegaal I.D., Lugli A. (2020). Are tumour grade and tumour budding equivalent in colorectal cancer? A retrospective analysis of 771 patients. Eur. J. Cancer.

[41] Jenkins M.A., Hayashi S., O’Shea A.M., Burgart L.J., Smyrk T.C., Shimizu D., Waring P.M., Ruszkiewicz A.R., Pollett A.F., Redston M. (2007). Pathology features in Bethesda guidelines predict colorectal cancer microsatellite instability: A population-based study. Gastroenterology.

[42] Roelands J., Kuppen P.J.K., Vermeulen L., Maccalli C., Decock J., Wang E., Marincola F.M., Bedognetti D., Hendrickx W. (2017). Immunogenomic Classification of Colorectal Cancer and Therapeutic Implications. Int. J. Mol. Sci..

[43] Picard E., Verschoor C.P., Ma G.W., Pawelec G. (2020). Relationships Between Immune Landscapes, Genetic Subtypes and Responses to Immunotherapy in Colorectal Cancer. Front. Immunol..

[44] Karpinski P., Rossowska J., Sasiadek M.M. (2017). Immunological landscape of consensus clusters in colorectal cancer. Oncotarget.

[45] Becht E., de Reynies A., Giraldo N.A., Pilati C., Buttard B., Lacroix L., Selves J., Sautes-Fridman C., Laurent-Puig P., Fridman W.H. (2016). Immune and Stromal Classification of Colorectal Cancer Is Associated with Molecular Subtypes and Relevant for Precision Immunotherapy. Clin. Cancer Res..

[46] Sirinukunwattana K., Domingo E., Richman S.D., Redmond K.L., Blake A., Verrill C., Leedham S.J., Chatzipli A., Hardy C., Whalley C.M. (2020). Image-based consensus molecular subtype (imCMS) classification of colorectal cancer using deep learning. Gut.

[47] Chen Y.P., Zhang Y., Lv J.W., Li Y.Q., Wang Y.Q., He Q.M., Yang X.J., Sun Y., Mao Y.P., Yun J.P. (2017). Genomic Analysis of Tumor Microenvironment Immune Types across 14 Solid Cancer Types: Immunotherapeutic Implications. Theranostics.

[48] Schumacher T.N., Schreiber R.D. (2015). Neoantigens in cancer immunotherapy. Science.

[49] Joost P., Bendahl P.O., Halvarsson B., Rambech E., Nilbert M. (2013). Efficient and reproducible identification of mismatch repair deficient colon cancer: Validation of the MMR index and comparison with other predictive models. BMC Clin. Pathol..

[50] Greenson J.K., Bonner J.D., Ben-Yzhak O., Cohen H.I., Miselevich I., Resnick M.B., Trougouboff P., Tomsho L.D., Kim E., Low M. (2003). Phenotype of microsatellite unstable colorectal carcinomas: Well-differentiated and focally mucinous tumors and the absence of dirty necrosis correlate with microsatellite instability. Am. J. Surg. Pathol..

[51] Smyrk T.C., Watson P., Kaul K., Lynch H.T. (2001). Tumor-infiltrating lymphocytes are a marker for microsatellite instability in colorectal carcinoma. Cancer.

[52] Alexander J., Watanabe T., Wu T.T., Rashid A., Li S., Hamilton S.R. (2001). Histopathological identification of colon cancer with microsatellite instability. Am. J. Pathol..

[53] Reynolds I.S., Furney S.J., Kay E.W., McNamara D.A., Prehn J.H.M., Burke J.P. (2019). Meta-analysis of the molecular associations of mucinous colorectal cancer. Br. J. Surg..

[54] Sandberg T.P., Stuart M., Oosting J., Tollenaar R., Sier C.F.M., Mesker W.E. (2019). Increased expression of cancer-associated fibroblast markers at the invasive front and its association with tumor-stroma ratio in colorectal cancer. BMC Cancer.

[55] Sandberg T.P., Oosting J., van Pelt G.W., Mesker W.E., Tollenaar R., Morreau H. (2018). Molecular profiling of colorectal tumors stratified by the histological tumor-stroma ratio—Increased expression of galectin-1 in tumors with high stromal content. Oncotarget.

[56] De Sousa E.M.F., Wang X., Jansen M., Fessler E., Trinh A., de Rooij L.P., de Jong J.H., de Boer O.J., van Leersum R., Bijlsma M.F. (2013). Poor-prognosis colon cancer is defined by a molecularly distinct subtype and develops from serrated precursor lesions. Nat. Med..

[57] Smit M. (2021). Tumour-stroma ratio outperforms tumour budding as biomarker in colon cancer: A cohort study. Int. J. Color. Dis..

[58] Kautto E.A., Bonneville R., Miya J., Yu L., Krook M.A., Reeser J.W., Roychowdhury S. (2017). Performance evaluation for rapid detection of pan-cancer microsatellite instability with MANTIS. Oncotarget.

