# Supplementary material for: Correlation of Immunological and Histopathological Features with Gene Expression-Based Classifiers in Colon Cancer Patients
Source: Int J Mol Sci. 2022 Oct 21;23(20):12707. doi: 10.3390/ijms232012707 (PMC9604175; doi:10.3390/ijms232012707)
Supplement: Supplementary file 1 [file ijms-23-12707-s001.zip › Supplementary Table S1.pdf]

	**CMS1**	**CMS2**	**CMS3**	**CMS4**	*p*-value
**pT2**	0 (0.0)	3 (4.1)	4 (10.0)	0 (0.0)	
**pT3**	39 (90.7)	65 (87.8)	29 (72.5)	56 (91.8)	0.041
**pT4**	4 (9.3)	6 (8.1)	7 (17.5)	5 (8.2)	

Table S1. Distribution of CMS subtypes per pathologic T-stage. P-value is derived from an overall comparison between subtypes.
