# Supplementary material for: Correlation of Immunological and Histopathological Features with Gene Expression-Based Classifiers in Colon Cancer Patients
Source: Int J Mol Sci. 2022 Oct 21;23(20):12707. doi: 10.3390/ijms232012707 (PMC9604175; doi:10.3390/ijms232012707)
Supplement: Supplementary file 1 [file ijms-23-12707-s001.zip › Supplementary Table S2.pdf]

	**CRIS-A**	**CRIS-B**	**CRIS-C**	**CRIS-D**	**CRIS-E**	*p*-value
**pT2**	3 (5.0)	0 (0.0)	3 (4.8)	1 (2.9)	0 (0.0)	0.875
**pT3**	50 (83.3)	31 (88.6)	55 (87.3)	31 (88.6)	22 (88.0)	
**pT4**	7 (11.7)	4 (11.4)	5 (7.9)	3 (8.6)	3 (12.0)	

Table S2. Distribution of CRIS subtypes per pathologic T-stage. P-value is derived from an overall comparison between subtypes.
