# Supplementary material for: Correlation of Immunological and Histopathological Features with Gene Expression-Based Classifiers in Colon Cancer Patients
Source: Int J Mol Sci. 2022 Oct 21;23(20):12707. doi: 10.3390/ijms232012707 (PMC9604175; doi:10.3390/ijms232012707)
Supplement: Supplementary file 1 [file ijms-23-12707-s001.zip › Supplementary Table S3.pdf]

	pT2	pT3	pT4	*p*-value
**TILs**				
None/low	6 (85.7)	141 (76.2)	18 (81.8)	0.661
Intermediate	1 (14.3)	30 (16.2)	4 (18.2)	
High	0 (0.0)	14 (7.6)	0 (0.0)	
**Mucus**				
≤50%	7 (100)	165 (89.2)	17 (77.3)	0.160
>50%	0 (0.0)	20 (10.8)	5 (22.7)	
**Mucus**				
<10%	4 (57.1)	131 (70.8)	13 (59.1)	0.416
≥10%	3 (42.9)	54 (29.2)	9 (40.9)	
**TSR**				
Stroma-low	7 (100)	125 (66.1)	13 (59.1)	0.130
Stroma-high	0 (0.0)	64 (33.9)	9 (40.9)	
**Tumor Budding**				
Low (<5)	7 (100)	150 (79.4)	19 (86.4)	0.439
Intermediate (5-9)	0 (0.0)	28 (14.8)	1 (4.5)	
High (≥10)	0 (0.0)	11 (5.8)	2 (9.1)	

Table S3. Correlation between histopathologic features and pathologic T-stage. TILs = tumor infiltrating lymphocytes, TSR = tumor-stroma ratio. P-values are derived from an overall comparison between pathologic T-stages.
