# Supplementary material for: Correlation of Immunological and Histopathological Features with Gene Expression-Based Classifiers in Colon Cancer Patients
Source: Int J Mol Sci. 2022 Oct 21;23(20):12707. doi: 10.3390/ijms232012707 (PMC9604175; doi:10.3390/ijms232012707)
Supplement: Supplementary file 1 [file ijms-23-12707-s001.zip › Supplementary Table S4.pdf]

	CMS1 vs. Rest			CMS2 vs. Rest			CMS3 vs. Rest			CMS4 vs. Rest		
	OR	*95% CI*	*p-value*	*OR*	*95% CI*	*p-value*	*OR*	*95% CI*	*p-value*	*OR*	*95% CI*	*p-value*
**TILs** High vs. Low	5.587	2.704-11.543	<0.001	0.256	0.109-0.605	0.001	2.240	1.056-4.751	0.033	0.287	0.115-0.715	0.005
**Mucus** >50% vs. ≤50%	3.152	1.302-7.628	0.008	0.032	0.002-0.530	<0.001	1.493	0.554-4.024	0.426	1.522	0.633-3.661	0.346
**Mucus** ≥10% vs. <10%	3.423	1.714-6.837	<0.001	0.076	0.026-0.219	<0.001	5.836	2.779-12.256	<0.001	0.597	0.301-1.184	0.138
**Stroma** High vs. Low	0.627	0.295-1.329	0.220	0.576	0.309-1.072	0.080	1.086	0.5286-2.233	0.822	2.327	1.263-4.289	0.006
**Budding** High vs. Low	1.354	0.606-3.028	0.459	0.636	0.300-1.352	0.238	0.409	0.137-1.222	0.100	2.040	1.009-4.126	0.045

Table S4. Odds Ratios with 95% confidence interval calculated as the likelihood for assignment into a specific CMS subtype if scored into the highest histopathologic category (i.e. TILs-high, Mucus >50%, Mucus ≥10%, Stroma-high and Budding-high). CMS = consensus molecular subtype, OR = odds ratio, CI = confidence interval, TILs = tumor infiltrating lymphocytes
