# Supplementary material for: Correlation of Immunological and Histopathological Features with Gene Expression-Based Classifiers in Colon Cancer Patients
Source: Int J Mol Sci. 2022 Oct 21;23(20):12707. doi: 10.3390/ijms232012707 (PMC9604175; doi:10.3390/ijms232012707)
Supplement: Supplementary file 1 [file ijms-23-12707-s001.zip › Supplementary Table S5.pdf]

	CRIS-A vs. Rest			CRIS-B vs. Rest			CRIS-C vs. Rest			CRIS-D vs. Rest			CRIS-E vs. Rest		
	OR	*95% CI*	*p-value*	*OR*	*95% CI*	*p-value*	*OR*	*95% CI*	*p-value*	*OR*	*95% CI*	*p-value*	*OR*	*95% CI*	*p-value*
**TILs** High vs. Low	2.507	1.280-4.908	0.006	2.347	1.079-5.105	0.028	0.272	0.109-0.677	0.003	0.533	0.194-1.460	0.215	0.644	0.209-1.984	0.441
**Mucus** >50% vs ≤50%	11.796	4.418-31.491	<0.001	0.971	0.312-3.025	0.959	0.087	0.012-0.661	0.003	0.197	0.026-1.508	0.084	0.132	0.008-2.246	0.085
**Mucus** ≥10% vs <10%	20.510	9.632-43.676	<0.001	0.741	0.326-1.683	0.473	0.164	0.067-0.405	<0.001	0.180	0.053-0.611	0.002	0.288	0.083-1.002	0.039
**Stroma** High vs. Low	0.803	0.422-1.525	0.502	0.644	0.284-1.456	0.288	0.990	0.532-1.843	0.976	1.861	0.893-3.882	0.094	1.134	0.475-2.706	0.777
**Budding** High vs. Low	0.464	0.194-1.111	0.080	2.247	0.998-5.059	0.046	1.130	0.544-2.348	0.744	1.057	0.427-2.617	0.904	0.777	0.252-2.397	0.660

Table S5. Odds Ratios with 95% confidence interval calculated as the likelihood for assignment into a specific CRIS subtype if scored into the highest histopathologic category (i.e. TILs-high, Mucus >50%, Mucus ≥10%, Stroma-high and Budding-high). CRIS = colorectal cancer intrinsic subtypes, OR = odds ratio, CI = confidence interval, TILs = tumor infiltrating lymphocytes
