# Supplementary material for: Correlation of Immunological and Histopathological Features with Gene Expression-Based Classifiers in Colon Cancer Patients
Source: Int J Mol Sci. 2022 Oct 21;23(20):12707. doi: 10.3390/ijms232012707 (PMC9604175; doi:10.3390/ijms232012707)
Supplement: Supplementary file 1 [file ijms-23-12707-s001.zip › Supplementary Table S6.pdf]

| | MSI-status | | |
|---|---|---|---|
| | *OR* | *95% CI* | *p-value* |
| **TILs**<br>High vs. Low | 6.571 | 3.102-13.920 | <0.001 |
| **Mucus**<br>>50% vs. ≤50% | 4.567 | 1.789-11.655 | 0.001 |
| **Mucus**<br>≥10% vs. <10% | 4.373 | 2.152-8.887 | <0.001 |
| **Stroma**<br>High vs. Low | 0.363 | 0.157-0.835 | 0.014 |
| **Budding**<br>High vs. Low | 0.968 | 0.406-2.309 | 0.968 |

**Table S6.** Odds Ratios with 95% confidence interval calculated as the likelihood for microsatellite instability if scored into the highest histopathologic category (i.e. TILs-high, Mucus > 50%, Mucus ≥ 10%, Stroma-high and Budding-high). MSI = microsatellite instability, OR = odds ratio, CI = confidence interval, TILs = tumor infiltrating lymphocytes
