# Supplementary material for: Correlation of Immunological and Histopathological Features with Gene Expression-Based Classifiers in Colon Cancer Patients
Source: Int J Mol Sci. 2022 Oct 21;23(20):12707. doi: 10.3390/ijms232012707 (PMC9604175; doi:10.3390/ijms232012707)
Supplement: Supplementary file 1 [file ijms-23-12707-s001.zip › Supplementary Table S7.pdf]

	CMS1	CMS2	CMS3	CMS4	*p*-value
**TILs combined with Mucus**					
TILs low or Non-mucinous	41 (95.3)	72 (100)	36 (92.3)	59 (98.3)	0.097
TILs high *and* Mucinous	2 (4.7)	0 (0.0)	3 (7.7)	1 (1.7)	
TILs low and Non-mucinous	13 (30.2)	65 (90.3)	22 (56.4)	46 (76.7)	<0.001
TILs high *or* Mucinous	30 (69.8)	7 (9.7)	17 (43.6)	14 (23.3)	
TILs low or Mucus <10%	34 (79.1)	72 (100)	29 (74.4)	59 (98.3)	<0.001
TILs high *and* Mucus ≥10%	9 (20.9)	0 (0.0)	10 (25.6)	1 (1.7)	
TILs low and Mucus <10%	7 (16.3)	61 (84.7)	10 (25.6)	41 (68.3)	<0.001
TILs high *or* Mucus ≥10%	36 (83.7)	11 (15.3)	29 (74.4)	19 (31.7)	
**TSR combined with Budding**					
Stroma-low or Budding low	40 (93.0)	69 (93.2)	36 (90.0)	51 (83.6)	0.257
Stroma-high *and* Budding high	3 (7.0)	5 (6.8)	4 (10.0)	10 (16.4)	
Stroma-low and Budding low	25 (58.1)	49 (66.2)	26 (65.0)	25 (41.0)	0.018
Stroma-high *or* Budding high	18 (41.9)	25 (33.8)	14 (35.0)	36 (59.0)	

Table S7. Correlation between different combinations of histopathologic features and CMS subtypes. TILs = tumor infiltrating lymphocytes, TSR = tumor-stroma ratio. P-values are derived from an overall comparison between subtypes.
