# Supplementary material for: Correlation of Immunological and Histopathological Features with Gene Expression-Based Classifiers in Colon Cancer Patients
Source: Int J Mol Sci. 2022 Oct 21;23(20):12707. doi: 10.3390/ijms232012707 (PMC9604175; doi:10.3390/ijms232012707)
Supplement: Supplementary file 1 [file ijms-23-12707-s001.zip › Supplementary Table S8.pdf]

	CRIS-A	CRIS-B	CRIS-C	CRIS-D	CRIS-E	*p*-value
**TILs combined with Mucus**						
TILs Low or Non-mucinous	54 (91.5)	34 (97.1)	62 (100)	34 (100)	24 (100)	0.034
TILs High *and* Mucinous	5 (8.5)	1 (2.9)	0 (0.0)	0 (0.0)	0 (0.0)	
TILs Low and Non-mucinous	24 (40.7)	19 (54.3)	55 (88.7)	28 (82.4)	20 (83.3)	<0.001
TILs High *or* Mucinous	35 (59.3)	16 (45.7)	7 (11.3)	6 (17.6)	4 (16.7)	
TILs Low or Mucus <10%	42 (71.2)	32 (91.4)	62 (100)	34 (100)	24 (100)	<0.001
TILs High *and* Mucus ≥10%	17 (28.8)	3 (8.6)	0 (0.0)	0 (0.0)	0 (0.0)	
TILs Low and Mucus <10%	10 (16.9)	16 (45.7)	50 (80.6)	26 (76.5)	17 (70.8)	<0.001
TILs High *or* Mucus ≥10%	49 (83.1)	19 (54.3)	12 (19.4)	8 (23.5)	7 (29.2)	

Table S8. Correlation between different combinations of histopathologic features and CRIS subtypes. TILs = tumor infiltrating lymphocytes. P-values are derived from an overall comparison between subtypes.
